# A1C as a Prognosticator of Perioperative Complications of Diabetes: A Narrative Review

**DOI:** 10.5152/TJAR.2021.854

**Published:** 2022-04-01

**Authors:** Raghuraman M. Sethuraman., Satyen Parida, Adinarayanan Sethuramachandran, Priyanka Selvam

**Affiliations:** 1 Department of Anaesthesiology, Sree Balaji Medical College & Hospital (BIHER), Chennai, India; 2 Department of Anaesthesiology, Jawaharlal Institute of Postgraduate Medical Education and Research, Puducherry, India; 3 Department of Anaesthesiology, Apollo Hospitals, Chennai, India

**Keywords:** Diabetes mellitus, glycated hemoglobin, hemoglobin A1c, hyperglycemia, mortality and morbidity, perioperative complications

## Abstract

Hemoglobin A1c (A1C) or glycated hemoglobin reflects the levels of blood glucose during the previous 8–12 weeks duration. It also helps us to diagnose diabetes in some cases, during the preoperative screening, who were initially missed out. Although the number of patients with diabetes undergoing various surgeries has increased many times, the role of A1C as a predictor for the complications during the perioperative phase remains intriguing. This could be due to various factors such as lack of best shreds of evidence, various cut-off levels of target A1C, variations of the patient population, presence of other comorbid conditions, and so on. This narrative review article presents the role of A1C as a reflector of perioperative adverse events in various surgeries and discusses the controversies surrounding it. We searched “PubMed Central” database with search criteria of “hemoglobin A1c, glycated hemoglobin, and perioperative complications” with publication date from January 01, 2010, to January 31, 2020, and found a total of 214 articles. We included only the relevant articles to our topic and added a few more articles that we found as “secondary references” from those articles to suit the structured headings of our narrative review and made it a total of fifty. To our knowledge, the majority of the studies published on this topic are of the “Retrospective analysis” type of study, besides no narrative review article available to date in the literature. We suggest that assessment of A1C levels preoperatively can be used as a routine practice for major procedures in patients with diabetes and for patients who have persistent high glucose values during preoperative screening regardless of whether a diagnosis of diabetes is established or not. We found that a cut-off of 8% is acceptable for the majority of the surgical procedures. However, it is better to have a cut-off of 7% or lower for procedures such as spine and joint replacement surgeries, cardiac surgeries, and so on. Further prospective studies involving a large population preferably with a multicenter design would provide us more clarity on this topic.

**Main Points**

- Although A1C has been used commonly to reflect the optimization of diabetes, its role as a predictor of perioperative complications remains intriguing.
- Majority of the studies published on this topic are of “Retrospective type” providing different conclusions.
- No consensus on the cut-off of A1C for preoperative stabilization has been reached so far.
- Prospective studies involving a large population preferably with a multicenter design can throw more clarity on this topic.

## Introduction

Hemoglobin A1C or glycated hemoglobin or simply termed as “A1C” for consistency^1^ can provide us with a scenario about blood glucose levels of the past 8-12 weeks duration. It also helps us to diagnose diabetes in some cases if blood glucose levels are equivocal. The World Health Organization (WHO) has given accordance to use A1C as a diagnostic tool of diabetes in 2011, provided strict quality tests with standardization to international reference values were in place that can provide accurate measurements.^2^ WHO has recommended 6.5% as a cut-off for diagnosing diabetes. Thus, A1C value of 6.5% or 48 mmol mol^−1^ (after the test is repeated) is considered as diagnostic of diabetes, while people with A1C values between 6.0% and 6.4% (42 and 47 mmol mol^−1^) are considered as having a high risk for developing diabetes warranting preventive measures.^2^ It must be noted that the values of A1C are provided in two types. The old method of expressing in percentage is as per the DCCT (Diabetes Control and Complications Trial) units; whereas, mmol mol^−1^ values, a newer method of expression, are provided as per the IFCC (International Federation of Clinical Chemistry) units. The significance of A1C can be understood from the fact that it was included as a parameter for the diagnosis of diabetes in the UK guidelines immediately following the recommendations by WHO.^2^ It is suggested that A1C should be estimated preoperatively in all patients subjected to major surgical procedures while it should be done in all patients with diabetes subjected to any elective surgical procedure.^2^ This would help to diagnose diabetes in some cases, which were originally missed out, besides making an impact on the scheduling of the surgery depending on the A1C value.^2^

As we are aware of the fact that perioperative hyperglycemia is strongly associated with unfavorable clinical consequences, we certainly need to take some measures to prevent them as much as possible. Preoperative A1C levels have been demonstrated to be a useful parameter to predict postoperative adverse events in various types of surgeries.^2–3^ Furthermore, preoperative A1C was found to have a strong prediction about mortality as well as morbidity following coronary artery bypass graft (CABG) surgeries regardless of preoperative diabetes status.^4^ Preoperative A1C levels also predict the intra-operative insulin sensitivity in cardiac surgeries, a factor that would play a major role in determining complications because intra-operative insulin resistance is associated with many complications regardless of preoperative diabetes status of the patients.^5^

The main advantage of A1C estimation is that it can be tested at any time in a day, and fasting is not mandatory. Also, good correlation doses exist between A1C and the postoperative blood glucose (BG) levels in both elective and emergency surgeries,^6–8^ as well as in patients who were not diagnosed to have diabetes.^9^ Therefore, A1C screening would help to identify persons at potential risk of developing postoperative hyperglycemia thereby reducing complications, besides diagnosing patients who did not have diabetes previously. Also, elevated A1C would result in the timely inclusion of an endocrinologist to monitor the further control of diabetes.

The main drawback of A1C estimation is that conditions such as hemoglobinopathies, certain anemias, and conditions that influence the life span of red blood cells (RBCs) would affect the A1C values.

This narrative review article presents the role of A1C as a reflector of perioperative adverse events in various surgeries with the available evidence and discusses the controversies surrounding it regarding health care costs and cut-off values. To our knowledge, the majority of the studies published on this topic are of “Retrospective analysis” type of study, besides no narrative review article is available to date in the literature.

## Role of A1C in Various Surgeries

### Cardiac Surgery

Optimization of patient care is realizable by settling the risks that may be confronted during cardiac surgeries in the preoperative period. Appropriate management of comorbidities, identifying the best intra-operative anesthetic care pathway, and watchful postoperative follow-up ensure curtailment of prognosticated risks. Hence, many scoring systems and some laboratory values are used preoperatively. Some of the parameters that are regularly assessed while assembling an anesthetic plan for the patient posted for surgery help us do a risk appraisal for the postoperative sequelae.

A1C is one of the most routinely researched investigations for the outcomes following cardiac surgery. However, scientific texts are divided on the topic of whether high A1C levels result in escalated mortality and morbidity rates, with reports proclaiming different outcomes.^4,10–11^

Poor regulation of blood sugar levels in the perioperative period during cardiac surgery has been reported to have negative postoperative effects.^12^ Furthermore, results have been communicated from other studies, suggesting that postoperative recovery is better in patients with diabetes who had strict preoperative blood sugar regulation.^13,14^ Reports appraising the risk factors linked with the results of cardiac surgery reinforce the belief that perioperative high blood sugar levels are independent predictors for postoperative morbidity and mortality.^15^ A retrospective analysis found that elevated A1C (>7%) was significantly associated with postoperative myocardial infarction only, while no significant difference was found regarding other complications such as renal failure, stroke, wound infection, and duration of intensive care unit (ICU) stay in patients undergoing coronary artery surgeries.^16^ It has been held that high A1C levels are a strong indicator of mortality and morbidity, independent of the patient’s earlier diabetes state and that the mortality risk escalates fourfold mainly in CABG when A1C levels are >8.6%. In other reports, it has been indicated that A1C is a strong predictor of morbidity and mortality in CABG and that A1C levels >7.03% amplify the risk of postoperative complications.^17,18^ In a study investigating long-term follow-up of CABG in patients with type 2 diabetes, it was concluded that mortality rate escalated when A1C levels were >9%, and morbidity and complications surged when the levels were >8.1%, though in the same study, mortality was not found to be linked with A1C levels in patients using insulin.^19^ Moreover, there are contrasting outcomes with respect to A1C level rise being predictive of the risk of cardiovascular events. When a risk appraisal was made for cardiovascular events in some reports, some of the outcomes were adjudged to be consequential in terms of A1C level rise, though A1C was not found to be predictive when multivariate regression statistical analysis was conducted apart from the other risk factors.^20,21^ Beattie et al.^22^ investigated perioperative cardiac biomarkers and proposed that A1C is not a useful appraisal criterion in the care pathway of surgical patients and that it is the biomarker of a system that cannot be modified as it reflects chronic control of diabetes. Nevertheless, preoperative A1C was the only biomarker to forecast the operative mortality following CABG surgeries implying preoperative medium-term control of diabetes is more valuable than intra-operative control of diabetes.^23^ Preoperative high A1C levels were found in 57% of patients who didn’t have diabetes and in 96% of patients with diabetes.^24^ In the light of these high rates, mortality and postoperative problems should have been predicted to be very high. However, studies showed that high A1C can be associated with postoperative major adverse events but not mortality.^11,25^ Furthermore, preoperative A1C predicts the risk of postoperative glycemic variabilities.^25^ Bardia et al.^26^ showed that postoperative problems and mortality were not correlated with A1C levels in patients coming in for only valve surgery. They found it striking that there was no link between the postoperative glycemic variability and the complications and stressed that it was divergent from the older results in CABG patients.

### Major Noncardiac Surgeries

Few reports have explored the interrelation connecting preoperative A1C level and noncardiac surgical sequel. Supplemental to this is the fact that the data from cardiac surgery is doubtless not suitable for extrapolation to noncardiac surgery patients.

Higher A1C level is, without doubt, a prognosticator of long-term complications of diabetes and is the main biological marker for glycemic regulation in diabetes. However, there is persistent doubt over whether long-standing hyperglycemia has an untoward effect on surgical results over and above acute perioperative hyperglycemia and whether care pathways that tackle raised A1C levels prior to surgery would boost clinical outcomes. As there is a paucity of data, different A1C cut-offs are used by clinicians.

A study by Underwood et al.^3^ conveys that A1C >8% is related to poor surgical results such as prolonged hospital length of stay (LOS). The authors submitted that coming up with a preoperative intervention to enhance glycemic regulation in patients with A1C values >8% may enhance surgical outcomes.

Hyperglycemia has been held responsible for the poor neutrophil phagocytic function, greater inflammation, oxidative stress, and hindered endothelial activity, aspects that could affect healing following surgery. Also, it is pertinent to note that long-term severe hyperglycemia (reflected by A1C) is likely to cause more severe damage than hyperglycemia noted during the perioperative period alone.^3^ High A1C level was shown to be equated with heightened risk for wound infection and acute kidney injury after gastric bypass surgery.^27^ High A1C level has also been shown to predict enhanced morbidity in various surgical procedures such as vascular surgery,^28^ total joint arthroplasty,^29^ and spine surgery.^30^ A meta-analysis published in 2017 based on six retrospective studies also found an association between high A1C and deep wound infection involving the prosthesis in total knee and hip arthroplasty procedures.^31^ Another retrospective study had observed that both preoperative and postoperative high A1C levels (>6.5% or 48 mmol mol^−1^), as well as hyperglycemia (>200 mg dL^−1^), were associated with greater rates of wound dehiscence among patients enlisted to the hospital for wound closure.^32^ A strong association was observed between high preoperative A1C levels (>7%) and surgical site infection (SSI) in thoracic and lumbar spine instrumentation procedures^33^ and sternum wound infection after CABG.^34^ In contrast to these studies, few studies found that there was no association between A1C and surgical outcomes. For instance, no link could be found between A1C level and any clinical outcomes in patients who had undergone laparoscopic gastric sleeve surgery.^35^ Similarly, a retrospective analysis in noncardiac surgery patients also did not find an association of preoperative A1C level as well hyperglycemia with surgical infections but substantiated higher rates of infections with hyperglycemia occurred during the first 24-hour postoperative period.^36^ A systematic review also observed that no definite association could be made between elevated A1C and adverse outcomes in various surgical populations. Nevertheless, the studies available for that systematic review published in 2016 were of a predominantly retrospective type that too with a smaller number of patients.^37^

After analyzing these studies carefully, we believe that both preoperative A1C levels and perioperative glucose levels should be considered equally important and treated accordingly, to prevent the adverse outcomes of any surgical procedure in patients with diabetes. Besides, we also suggest that more caution should be exercised in patients who have elevated A1C levels regardless of the diagnosis of diabetes made in the preoperative period.

## Role of A1C in Health Care Costs

Higher incidences of postoperative complications in patients with diabetes could lead to greater utilization of resources for these patients resulting in higher health care costs. For instance, deep wound infection following a joint replacement would certainly increase the LOS in the hospital thereby increasing the costs considerably. A recent meta-analysis (albeit based on six retrospective studies) has found an association between high A1C, hyperglycemia, and prosthetic infection following joint replacement surgeries.^31^ Similarly, SSI in major orthopaedic procedures such as spine instrumentation,^33^ wound infection of sternum following coronary artery surgeries^34^ can certainly prolong the LOS. Thus, measures chosen for reducing postoperative adverse outcomes in patients with diabetes may lead to better surgical results, decrease LOS, more logical distribution of health care facilities and resources, all resulting in lowering of health care costs significantly. However, the reports available for the association between A1C and LOS are conflicting with some studies observing that elevated preoperative A1C levels prolong LOS in non-cardiac procedures,^3^ and in cardiac procedures,^38^ while other studies concluding with no impact in cardiac surgeries.^16, 39^ We believe that this could be due to various factors such as different cut-offs of A1C, different number of days to consider prolonged LOS, difference in study populations, and so on. With the background of this confusing picture, it is not surprising that the attention is placed on single BG estimations on the day of operation or perioperative control of glucose even now. We do not deny the fact that evidence does exist for the association between acute rise in BG during and after surgery and poor surgical outcomes. However, we believe that it would be better if A1C levels are also taken into account in addition to perioperative BG levels. A recently published retrospective study corroborates this statement as it concluded that A1C can be used as a tool besides the fasting BG to predict postoperative complications.^40^ Because A1C predicts the postoperative glucose levels and some complications fairly well, it could result in lesser LOS in the hospital, consequently reducing the health care costs. Besides, the more vigilance exercised in patients with elevated preoperative A1C levels could result in lesser readmissions.^41^

### What Is the Ideal Cut-Off of A1C?

The ideal cut-off that can be applied for preventing perioperative complications in various types of surgical procedures is a major controversy surrounding A1C, although the cut-off values for diabetes and pre-diabetes states are clearly defined. The American Diabetes Association (ADA) has specified that A1C values of ≥6.5% (48 mmol mol^−1^) and 5.7–6.4% (39–47 mmol mol^−1^) are considered as diabetes and pre-diabetes states, respectively. As per the ADA guidelines, the test should be performed in a laboratory using a method that is NGSP (National Glycohemoglobin Standardization Program) certified and standardized to the DCCT assay.

Underwood et al.^3^ retrospectively studied the relationship between various preoperative A1C levels (≤6.5%, 6.5%–8%, 8%–10%, >10%) and clinical outcomes in patients with diabetes undergoing noncardiac surgery, as there was a general lack of data and application of different A1C cut-offs. They concluded that A1C levels < 6.5% and >8% were associated with enhanced LOS in hospital following operation and also A1C is a prognosticator of LOS in hospital, regardless of BG on the day of operation. They also concluded that the enhanced LOS in hospital in patients with A1C levels <6.5% could be related to the gravity of their illness, as suggested by their CCI (Charlson Comorbidity Index).

Hudson et al.^42^ in a retrospective observational study looking at A1C levels and outcomes in nondiabetic elective cardiac surgical patients concluded that an elevated preoperative A1C level (>6.0%) was independently associated with significantly higher early mortality risks (53% per % increase in A1C). They also suggested that A1C might be a valuable screening parameter to identify high-risk cardiac surgery patients who did not have diabetes.

The French Society of Anaesthesia and Intensive Care Medicine (SFAR) and the French Society for the Study of Diabetes (SFD), while making a joint position statement on perioperative diabetes management in 2018, recommended that elective surgery might be postponed if A1C levels are more than 9% as these patients are vulnerable to acute metabolic complications. Similarly, postponement of surgery is warranted if A1C is less than 5% as these patients are potential candidates for recurrent severe hypoglycaemias.^43^ The Joint British Diabetes Societies in its March 2016 revised guidelines recommended that all measures should be undertaken to achieve the A1C level <8.5% (69 mmol mol^−1^) before elective surgery.^44^ Despite the lack of consensus regarding cut-off of A1C that can be considered as safe to proceed with surgery, A1C value of >8% increases the risk for perioperative complications, as per the preoperative optimization program published in 2018.^45^

A recent review article states that adverse surgical outcomes happen when A1C levels are elevated and this association begins even at 6% (43 mmol mol^−1^), a range that is considered as pre-diabetes.^46^ According to a research article published recently, A1C level of more than 8% is a significant factor for SSI, which in turn would lead to enhanced LOS, costs, and mortality.^47^ Similarly, a lower number of re-interventions required during twelve months postoperative period in addition to better healing of ulcerations after six months in patients with A1C levels of ≤8.0% when compared to A1C levels of >8% in endovascular treatment for peripheral arterial diseases as per the observations of a recently published prospective study.^48^ Long-term complications were worse in patients who have undergone CABG with higher A1C levels (>8%), although no difference was observed regarding short-term outcomes and death as per the observations of a multicenter study published recently.^49^ Preoperative A1C levels of ≥5.6% (Pre-diabetes cut-off) have been significantly associated with acute kidney injury following CABG according to a recent retrospective analysis.^50^

### Limitations of A1C

The application of A1C in clinical practice has some limitations that necessitate the clinicians to exercise some cautions and/or modifications for accurate interpretation of the values. As already mentioned in the introduction, clinical conditions having an impact on the life span of RBCs, certain anemias, and hemoglobinopathies would affect A1C values. Because of the shortening of the life span of RBCs in pregnancy, A1C should be measured more frequently than in nonpregnant patients. Also, racial differences might play a role in the estimations of A1C. Fructosamine or glycated albumin (GA) can be considered as an alternative to A1C in clinical situations where A1C estimations would give an inaccurate picture of the BG values. Also, these two biomarkers reflect the BG levels of the past 1–3 weeks.

## Conclusions

With the available evidence (the majority being retrospective type), A1C can be considered as a predictor of perioperative glucose levels at least, which would certainly guide us to plan the control of diabetes accordingly. Hence, we suggest that assessment of A1C levels preoperatively can be used as a routine practice for major procedures in patients with diabetes as well as for patients who have persistent high glucose values during preoperative screening regardless of whether the diagnosis of diabetes is established or not. We also suggest that a cut-off of 8% is acceptable for the majority of the surgical procedures. However, it is better to have a cut-off of 7% or lower for procedures such as spine and joint replacement surgeries, cardiac procedures, and so on. Further prospective studies involving a large population preferably with a multicenter design would provide us more clarity on this topic.
